# Understanding patient preference in prosthetic ankle stiffness

**DOI:** 10.1186/s12984-021-00916-1

**Published:** 2021-08-25

**Authors:** Tyler R. Clites, Max K. Shepherd, Kimberly A. Ingraham, Leslie Wontorcik, Elliott J. Rouse

**Affiliations:** 1 Department of Mechanical Engineering, University of Michigan, Ann Arbor, MI 48109 USA; 2 Robotics Institute, University of Michigan, Ann Arbor, MI 48109 USA; 3 Department of Biomedical Engineering, Northwestern University, Evanston, IL 60208 USA; 4 Shirley Ryan Ability Lab, Chicago, IL 60611 USA; 5 Department of Physical Medicine and Rehabilitation, Michigan Medicine, University of Michigan Orthotics and Prosthetics Center, Ann Arbor, MI 48104 USA; 6 Neurobionics Lab, University of Michigan, Ann Arbor, MI 48109 USA

## Abstract

**Background:**

User preference has the potential to facilitate the design, control, and prescription of prostheses, but we do not yet understand which physiological factors drive preference, or if preference is associated with clinical benefits.

**Methods:**

Subjects with unilateral below-knee amputation walked on a custom variable-stiffness prosthetic ankle and manipulated a dial to determine their preferred prosthetic ankle stiffness at three walking speeds. We evaluated anthropomorphic, metabolic, biomechanical, and performance-based descriptors at stiffness levels surrounding each subject’s preferred stiffness.

**Results:**

Subjects preferred lower stiffness values at their self-selected treadmill walking speed, and elected to walk faster overground with ankle stiffness at or above their preferred stiffness. Preferred stiffness maximized the kinematic symmetry between prosthetic and unaffected joints, but was not significantly correlated with body mass or metabolic rate.

**Conclusion:**

These results imply that some physiological factors are weighted more heavily when determining preferred stiffness, and that preference may be associated with clinically relevant improvements in gait.

**Supplementary Information:**

The online version contains supplementary material available at 10.1186/s12984-021-00916-1.

## Introduction

The field of assistive robotics has become remarkably adept at tailoring device design and control to maximize application-specific performance. Because new designs are validated according to a limited set of metrics, the field’s understanding of what “works” is inherently tied to the details of those metrics. Even if resultant design biases are not intentional, the integration of knowledge from the literature and decades of shared experience inevitably drives design in the direction of historical and current measures of success. Some recent approaches, such as human-in-the-loop optimization, go as far as making objective-driven design an explicit part of the tuning process; in these approaches, system parameters are manipulated with the express goal of minimizing a carefully-constructed but highly simplified cost function [1–3]. In objective-driven design, whether explicit or not, it is crucial to select criteria that capture the spectrum of relevant important outcomes; otherwise, we risk sacrificing performance according to any metrics that are omitted.

Meaningful efficacy criteria are especially important in the design, evaluation, and prescription of clinical assistive technologies. Despite their promise for restoration of normative gait following amputation, injury, or other limb pathologies, robotic lower-extremity prostheses and orthoses have yet to see widespread adoption. Recent advancements in mechatronic hardware [4–7] and control paradigms [8–12] have made possible a transformation in the treatment of limb pathology, enabling a paradigm that takes full advantage of robotic limb components. As clinical practice progresses with these advancements, it will be necessary to demonstrate the value of each new assistive technology to payers, providers, and users. Although payers and providers are likely to be compelled by quantitative representations of ambulatory ability and overall performance, users may rely on different evaluation tools when choosing whether to make prescribed devices an intimate part of their life. As such, in addition to common biomechanical and metabolic outcome measures, it is prudent to assess assistive technologies in ways that promote noticeable improvements in wearer perception of their own performance [13–16].

Unfortunately, practical constraints—including equipment requirements, measurement time, and analytical overhead—make it difficult to integrate and interpret efficacy criteria that are derived from several simultaneous metrics. This is especially true at the prescription stage, as clinicians do not typically have access to the resources available in the research setting, such as motion capture, force plates, or metabolic monitors. In addition, many patient-specific priorities (e.g. comfort, stability, muscle fatigue) can be difficult to quantify with certainty. As a result of these challenges, researchers tend to motivate and validate their new devices with a narrow set of metrics; criteria that have been used include muscle activation [17–19], joint work [20, 21], limb power [22], self-selected walking speed [23, 24], peak moments [25, 26], range of motion [27, 28], kinematic symmetry [29, 30], metabolic cost [22, 31–33], and extensive patient-reported surveys [34–37]. Furthermore, these factors may provide competing indications of “optimal” device behavior.

We propose to introduce user preference as an alternative criterion to assess efficacy for designing, tuning, and prescribing prostheses, orthoses, and exoskeletons. It stands to reason that user input should play a substantial role in characterizing an assistive device’s clinical impact, irrespective of underlying pathology. In fact, patients are regularly asked to anecdotally describe their feelings of comfort and stability when walking with a new prosthetic leg, as part of the current clinical prescriptive process [38]. Users likely benefit from their direct perception of the device’s interaction with their body, derived from the vast array of physiological information available to them. As such, their preference likely encodes many of the physiological and biomechanical factors that contribute to normative gait. In addition, preference is inherently specific to the population from which it is measured, which increases its applicability as an efficacy criterion across pathologies, and drives device development toward solutions that meet patient needs. In light of these benefits, preference has emerged in the research setting as a potential indicator of device efficacy [39–41], and has recently been used in combination with human-in-the-loop optimization to tune the behavior of complex wearable mechatronic systems [42, 43].

Although user feedback currently plays an informal role in clinical prescription, two key roadblocks have prevented the formal incorporation of user preference as a measure of efficacy for clinical assistive technologies. The first of these roadblocks is practical: until recently the field has lacked rigorous, repeatable methodologies to quantify user preference of control parameters for wearable robotics [39, 43, 44]. To meet this need, we propose a simple user-driven measurement paradigm, which we have shown enables consistent identification of preference in a continuous, one-dimensional parameter landscape [39]. Our approach does not require expensive equipment, and enables rapid searching of a parameter space in both the laboratory and clinical settings. The second key hurdle is that the field’s understanding of preference is not yet sufficient to enable robust interpretation by researchers, payers, and providers. For instance, it is not known how preferred device parameters relate to typical clinical metrics of performance, such as the 10 Meter Walk test (10MWT), or whether users prefer parameters that are “good for them” long term [45]. If user-preferred parameters align with these more well-known metrics, providers may place more trust in the feedback they receive from patients.

We also do not know what factors users might prioritize when developing preference, or what measurable quantities correlate with preference in a way that would permit objective-driven design, control, and fitting. If, for instance, consistent across-user trends exist between preference and a simple biomechanical variable (e.g., prosthesis push-off work), designers could target improvement of this variable, and clinicians could focus more time honing device parameters towards this variable. More broadly, these gaps in knowledge create uncertainty around how preference should be used in the assessment of assistive technologies, and may have led the field to underutilize user preference as an indicator of success.

To address these deficiencies in understanding, we leveraged a quasi-passive prosthetic ankle–foot in a systematic evaluation of user preference with respect to a single, intuitive control parameter. The Variable Stiffness Prosthetic Ankle (VSPA) Foot has the unique ability to rapidly adjust the stiffness of its ankle joint during continuous walking. We gave control of this parameter to each of seven persons with unilateral below-knee amputation, and asked them to identify their preferred stiffness during treadmill walking at different speeds. We then evaluated gait biomechanics and metabolic expenditure at several stiffness values above and below the preferred stiffness. Our objectives in this analysis were to (i) understand how behavioral and anatomical factors, such as walking speed and body mass, affect preference; (ii) quantify how kinematic, kinetic, metabolic, and performance outcome measures vary across prosthetic ankle stiffness levels; and (iii) explore biomechanics factors that may be important to the user by identifying kinematic, kinetic, or metabolic descriptors that have local minima or maxima at or near the preferred stiffness.

## Methods

### Study design and subject selection

The primary hypotheses investigated in this study are that (i) preferred prosthetic ankle stiffness varies significantly across walking speeds, and (ii) kinematic, kinetic, metabolic, and performance outcome measures vary significantly across prosthetic ankle stiffness values, with at least one measure having a local optimum at or near the preferred stiffness. The experiments described herein were designed to highlight specific measurable factors that contribute to user preference. This was a crossover study design, in which each subject was exposed to several experimental conditions (walking speeds and prosthetic stiffnesses). Seven subjects (6M, 1F) with unilateral below-knee amputation participated in the study. Subjects were recruited from the University of Michigan Orthotics and Prosthetics Center, and constitute a representative sample of different body masses (range 58.6 to 99.2 kg) of persons with amputation. All subjects were community ambulators (K3 activity level or higher) without complicating lower-extremity injury, who regularly use conventional passive prostheses. All experiments were carried out with informed consent at the University of Michigan, with approval from the Institutional Review Board of the University of Michigan Medical School (IRBMED).

### VSPA-Foot

**Fig. 1:**
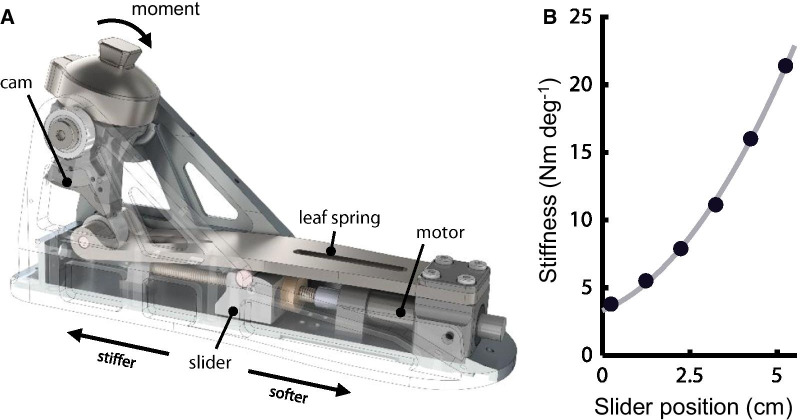
VSPA Foot characterization. **A** VSPA Foot under load. The VSPA Foot has a mechanically-programmable torque–angle curve shape, determined by the shape of the cam. An onboard motor adjusts VSPA stiffness during the swing phase of gait. **B** VSPA dorsiflexion stiffness versus slider position. Light line shows a second-order polynomial fit to experimental characterization data (plotted points). Only dorsiflexion stiffness is shown; the device is three times more compliant in plantarflexion

This study was performed with the Variable-Stiffness Prosthetic Ankle (VSPA) Foot, a quasi-passive lower-extremity prosthesis that supports complete specification of the ankle joint’s torque–angle relationship [46, 47]. The device features a mechanically-programmable cam-based transmission, which enables offline customization of torque–angle curve shape, as well as a motorized drive system for continuous, step-to-step modulation of ankle joint stiffness (Fig. 1A, Additional file 2: Movie S1). This quasi-passive design strikes a balance between the broader capabilities of powered prostheses, which come with the cost of added mass and diminished ability to smoothly and repeatably emulate passive mechanics, and the limited functional capacity of conventional passive prosthetic feet. The VSPA Foot’s dominant mechanics are passive, meaning that all restorative torque about the joint comes from deformation of lightweight energy-storing elements within the device, and that there is a defined functional relationship between joint angle and joint torque. Because the onboard motor that modulates these passive mechanics is active only during the swing phase of gait, while the foot is in the air and under negligible load, only a small motor is needed; the mass of the whole drive system is less than 50 g, resulting in an overall system mass that is 45% lower than the only commercially available active prosthetic ankle–foot (emPower, Ottobock, Duderstadt, Germany).

Despite its relatively low-mass design, the VSPA Foot’s fully customizable torque–angle relationship enables the device to closely approximate the passive components of biological ankle–foot mechanics [46, 48]. Step-to-step modulation of joint stiffness adds the unique ability to adapt joint stiffness for different gait tasks, including walking speeds, stair ascent and descent, and ground slope. In this study, our objective was to explore potential drivers of preferred stiffness at different walking speeds. To keep our analysis consistent with prior work in the field, we selected a cam for our experiments that produces a linear torque–angle shape, with a plantarflexion stiffness equal to 33% of dorsiflexion stiffness [39], which was determined in pilot experiments to provide soft weight acceptance without foot-slap at the preferred dorsiflexion stiffness. All stiffness values reported in the manuscript correspond explicitly to the dorsiflexion stiffness. The VSPA Foot used in this study contains small modifications from the device as previously described [39]. As such, the torque–angle relationship (Fig. 1B) was characterized for this new device on a custom rotary dynamometer, using the protocol described previously [46]. Minimum and maximum achievable stiffness values were 3.4 Nm deg^−1^ and 23.3 Nm deg^−1^ respectively, producing a total range of 5.9 times the minimum stiffness.

### Prosthesis fitting and training

Prior to donning the VSPA Foot, each subject’s self-selected overground walking speed was measured as they walked on their daily-use prosthesis (10 Meter Walk Test). A certified prosthetist then disconnected each subject’s daily-use prosthesis from their prosthetic socket, and affixed the VSPA Foot in its place; each subject’s daily-use socket was used for all study experiments. Alignment was adjusted according to standard clinical practice, with VSPA stiffness set to a nominal value based on each subject’s weight [39]. After fitting and alignment, we encouraged each subject to spend time acclimating to overground walking on the VSPA Foot at different stiffness values. This acclimation continued until each subject indicated that they were comfortable walking on the prosthesis (approximately 10–15 min). Once subjects were acclimated to the device, we reset VSPA stiffness to the nominal value, and identified self-selected treadmill walking speed.

### Preference identification

**Fig. 2:**
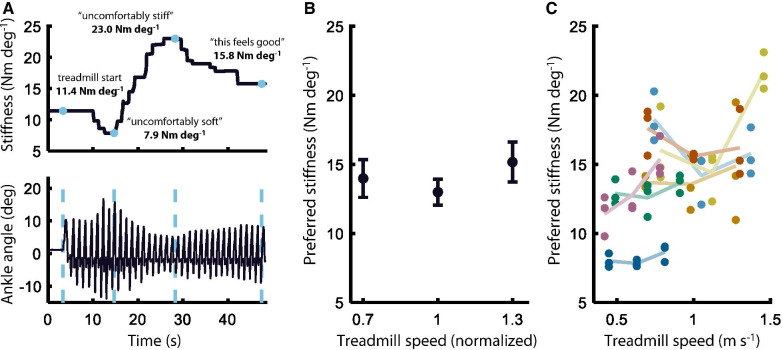
Preference identification. **A** Representative preference identification trial. Subjects used a mechanical dial to directly control VSPA stiffness while walking on a treadmill. Subjects were encouraged to explore the full range of stiffness values before identifying their preferred stiffness. **B** Inter-subject average preferred stiffness at different treadmill speeds. A LMEM showed a significant second-order relationship (*p* = 0.0046). Treadmill speed is normalized to each subject’s self-selected treadmill speed. Error bars show Standard Error of the Mean (SEM). **C** Individual preference selections. Six of the seven subjects preferred lower VSPA stiffness at their self-selected treadmill speed, compared to speeds above or below the self-selected speed. Each subject’s selections are shown in a different color. Light lines connect each subject’s mean preferred stiffness at each treadmill speed

Subjects were instructed to use a sensorized dial to identify their preferred stiffness during treadmill walking at three different speeds (+ 0%, ± 30% deviation from self-selected treadmill speed). The dial, which directly controlled VSPA stiffness via a microcomputer, was designed with infinite rotation and no absolute reference. This made it impossible for subjects to rely on direct external indicators of VSPA stiffness while identifying their preference. The dial could be freely rotated beyond the minimum and maximum of VSPA stiffness; however, the VSPA Foot saturated at these extrema, such that any supramaximal changes to the dial were ignored by the controller. This method of adjustment converges faster to the user’s preference than the two-alternative forced choice methods we have previously implemented [45, 49], and produces slower and more predictable (and thus safer) stride-to-stride adjustment of mechanics. Although subjects were free to rotate the dial throughout the gait cycle, stiffness was only actively adjusted, to match the value indicated by the dial’s position, during the swing phase of gait. During each preference identification trial, subjects were first encouraged to explore the full range of possible VSPA stiffness values, from “uncomfortably soft” to “uncomfortably stiff,” (with order of exploration freely chosen by the subject) before searching for and indicating their preferred stiffness (Fig. 2A). At the conclusion of each trial, we remotely set VSPA stiffness to a random value, within 25% of the most-recently-indicated preferred value. This protocol was repeated three times for each of the three treadmill speeds (nine total trials), in pseudo-random order; pre-generated trial orders were randomly assigned to different subjects, to ensure a balanced distribution in a limited sample size.

### Metabolic rate

Metabolic rate and gait biomechanics were recorded simultaneously at different VSPA stiffness values and treadmill speeds. These trials were carried out during a second experimental session, scheduled at least 2 days after the preference identification experiment. To ensure continuity across trial days, each subject’s prosthetic alignment was carefully preserved between sessions. On the morning of the metabolic trials, subjects were instructed to eat a light breakfast, then to refrain from eating for at least 2 h prior to the start of data collection. Metabolic rate was calculated from instantaneous measurements of inspired oxygen and expired carbon dioxide, measured via a portable pulmonary gas exchange unit (COSMED K5, Rome, Italy).

Each subject’s resting metabolic rate was first collected during 5 min of quiet standing. This resting rate was subtracted from all subsequent metabolic measurements, yielding net metabolic rate. Net metabolic rate was measured on the daily-use prosthesis, while each subject walked for 6 min at the self-selected, slow (− 30% self-selected), and fast (+ 30% self-selected) treadmill speeds, in pseudorandom order. Once these baseline net rates were established, the study prosthetist removed the daily-use prosthesis from each subject’s socket, affixed the VSPA Foot in its place, and gave each subject approximately 10 min to re-acclimate to the device. Subjects then returned to the treadmill and walked at each treadmill speed (self-selected, slow, fast), while VSPA stiffness was set to each of five different stiffness values, in random order. The experimental VSPA stiffness values for these trials were selected to be multiples (+ 0%, ± 15%, ± 30%) of each subject’s average preferred stiffness for the given treadmill speed, as measured during the preference identification trials. VSPA stiffness was changed remotely between trials without interruption to subject gait. Stiffness modulation occurred during the swing phase, and was always completed within two steps.

For each treadmill speed, all five trials were completed as a single, consecutive block. Remote, near-instantaneous changes to VSPA stiffness ensured a step-change in metabolic rate between trials in a single block. This experimental design enabled us to estimate steady-state metabolic rate using dynamic modeling techniques, rather than waiting for each subject’s metabolic rate to reach steady-state after each stiffness change [50]. Specifically, steady-state metabolic rate for each stiffness was calculated from a first-order dynamic model fit to the net metabolic rates from each trial within a given speed [1, 50, 51]. This analysis assumes that the breath-by-breath measurements are characterized by a first-order linear model,

$$\dot{y}\left(t\right)=\frac{1}{\tau }\left(x\left(t\right)-y\left(t\right)\right),$$

where $x(t)$ is the metabolic rate that would be achieved at steady-state, $y(t)$ is the breath measurement recorded at time $t$, and $\tau$ is the time constant for the model. To solve for steady-state metabolic rate, the first order model was discretized and the pseudoinverse of a constructed matrix was employed. For a detailed derivation of this analysis, we direct the reader to [1] and [2]. In each subject’s first block (i.e., the randomly-selected first of the three treadmill speeds), the first three trials each lasted 4 min, and the remaining two trials lasted 3 min. In the second and third blocks, the first trial lasted 4 min, and the remaining four trials each lasted 3 min. The 4-min trials were collected to allow us to estimate subject-specific time constants for the dynamic modeling, but we instead opted to use an average time constant of 42 s (determined in [50]) to be consistent with previous literature [2]. Leveraging the dynamic model, total treadmill walking time was reduced by approximately 20% compared to steady-state methods, which made it feasible to collect all metabolic data in a single day. Zhang et al. used a similar protocol but with an ankle exoskeleton, and characterized a 2.1% median error when comparing model-fit metabolic estimates after the first 3 min to their results for the full 6-min trials [2].

### Lower-extremity biomechanics

Biomechanics data were collected simultaneously with metabolic rate, during the second trial day. A standardized set of 36 reflective spherical markers were affixed to each subject’s pelvis, legs, and feet according to a modified Helen Hayes model [52]. Lower-extremity kinematics were recorded at a sampling rate of 100 Hz using a 17-camera motion capture system (Vicon Motion Systems Ltd, Oxford, UK). Markers were labelled automatically in Nexus software (Vicon), and manually checked for accuracy. Any gaps in marker trajectories were filled with spline, cyclic, or neighbor-tracking algorithms. Ground reaction force (GRF) data were measured independently for each leg by two force plates in the instrumented treadmill (Bertec, Columbus, OH). GRF signals from the treadmill were amplified, sampled at 1 kHz, digitized, and recorded. Force signals were digitally synchronized with the kinematic motion-capture system using Nexus software (Vicon). GRF and marker data were transformed to be consistent with the ground coordinate frame used in OpenSim (Stanford University, Palo Alto, CA) [53, 54], and then digitally low-pass filtered using a forward-reverse fourth-order Butterworth filter with a 15 Hz cutoff frequency (Matlab, MathWorks, Natick, MA, USA).

Inverse kinematic and dynamic analyses were performed in OpenSim v4.0 [55], using the *gait10dof18musc* model. We modified the model to fit our experimental marker set, and changed affected-side scaling parameters to (i) use femoral segment length estimates from the sound side, which avoids relying on socket markers to estimate prosthetic knee joint center, (ii) allow the location and orientation of the modeled VSPA Foot’s joint axis to differ from that of the unaffected ankle joint, and (iii) reflect the mass properties of the prosthetic device and socket. Bone dimensions were scaled for each subject using OpenSim’s Scale Tool, based on the subset of markers placed over easily-identifiable anatomical landmarks. Mass properties for each segment were scaled relative to body mass, and segment inertias relative to both mass and limb segment lengths. Model marker locations were adjusted for each subject based on a static pose trial, in which the subject stood with feet at shoulder width. We determined joint angles during walking using OpenSim’s Inverse Kinematics Tool, which constrains joint motion to modeled joint kinematics, and seeks to minimize the squared errors in resultant marker trajectories [54]. We then used OpenSim’s Inverse Dynamics Tool to estimate net joint moments from the inverse kinematics results and the measured ground reaction forces. Kinetic and kinematic trajectories, including joint angles, net joint moments, and net joint powers, were calculated for each stiffness and speed. Net power at each joint was calculated by multiplying the net joint moment by the joint angular velocity, which was calculated as the discrete time derivative of joint angle.

All subsequent analyses of gait biomechanics data were performed in Matlab. Kinematic and kinetic trajectories were split into gait cycles based on GRF data, and time normalized to percent gait cycle. Stance phase was also identified as the portion of each gait cycle during which there was a sustained, positive vertical GRF. Gait cycles were excluded from analysis if the GRF data showed that stance-phase foot plate was not isolated to a single force plate. From these gait-cycle-normalized trajectories, the biomechanical descriptors in Table 1 were calculated for each subject, for each stiffness and speed, as the descriptor’s average value across all stance phases from that stiffness and speed.

**Table 1:** Biomechanical descriptors and their trends with stiffness

	*p*_L_	*p*_Q_	v_lb_	v_ub_
Hip				
Cross-leg diff. in max. hip height Cross-leg difference in stepwise maximum hip height	0.666	0.289	−	−
Cross-leg RMS diff. hip angle Cross-leg root mean squared difference of stepwise hip angle trajectories	0.421	0.759	−	−
Cross-leg RMS diff. hip moment Cross-leg root mean squared difference of stepwise hip moment trajectories	0.066	0.950	−	−
Pelvic tilt variance Pelvic tilt variance	2e-4*	0.039	- 2.0	69.1
Knee				
Affected knee ROM Affected-side knee range of motion	2e-4*	0.177	−	−
Affected knee peak flex. moment Affected-side peak knee flexion moment	6e-13*	0.381	−	−
Affected knee peak ext. moment Affected-side peak knee extension moment	0.002	0.508	−	−
Unaffected knee ROM Unaffected-side knee range of motion	0.653	0.930	−	−
Unaffected knee peak early flex. angle Unaffected-side peak knee flexion angle in the first half of stance phase	2e-16*	0.426	−	−
Unaffected knee peak early ext. moment Unaffected-side peak knee extension moment in the first half of stance phase	2e-8*	8e-3	8.2	72.0
Cross-leg RMS diff. knee angle Cross-leg root mean squared difference of stepwise knee angle trajectories	0.451	0.092	−	−
Cross-leg RMS diff. knee moment Cross-leg root mean squared difference of stepwise knee moment trajectories	0.546	0.110	−	−
Cross-leg diff. in peak knee ext. moment Cross-leg difference of stepwise peak knee extension moments	0.036	0.584	−	−
Ankle				
Affected ankle peak DF angle Affected-side peak (prosthetic) ankle dorsiflexion angle	3e-33*	0.020	22.4	255
Affected ankle ROM Affected (prosthetic) ankle range of motion	3e-37*	5e-3	40.2	215
Affected ankle peak PF moment Affected-side peak (prosthetic) ankle plantar flexion moment	1e-10*	0.019	7.2	100
Affected ankle peak power Affected-side peak (prosthetic) ankle power	7e-18*	0.334	−	−
Unaffected ankle ROM Unaffected-side ankle range of motion	0.576	0.863	−	−
Unaffected ankle peak PF moment Unaffected-side peak ankle plantar flexion moment	0.131	0.598	−	−
Cross-leg RMS diff. ankle angle Cross-leg root mean squared difference of stepwise ankle angle trajectories	0.182	9e-4*	- 18.0	3.95
Cross-leg RMS diff. ankle moment Cross-leg root mean squared difference of stepwise ankle moment trajectories	4e-4*	0.535	−	−
Cross-leg diff. in peak ankle PF moment Cross-leg difference of stepwise peak ankle plantar flexion moments	1e-5*	0.167	−	−
Cross-leg diff. in peak ankle power Cross-leg difference of stepwise peak ankle powers	0.099	0.991	−	−
Other				
Affected step prop. of total stride duration Affected-side step duration as a proportion of total stride duration	0.017	0.451	−	−
Affected step prop. of total stride distance Affected-side step distance as a proportion of total stride distance	0.276	0.965	−	−

Twenty-five descriptors were evaluated for first- and second-order fit with preference-normalized VSPA stiffness. The table shows *p*-values for the linear (*p*_L_) and quadratic (*p*_Q_) terms of the LMEM fit to each descriptor. For descriptors with *p*_Q_ < 0.05, the lower (v_lb_) and upper (v_ub_) bounds on the 95% CI for the vertex stiffness (percent deviation from preferred) are also shown.

*Indicates significance at the α = 0.05 level (Bonferroni correction for 25 tested hypotheses)

### Self-selected overground walking speed

In this part of the experiment, we measure the effect of VSPA stiffness on self-selected overground walking speed; note that this is the experimental inverse of the preference identification trials (described above), in which we measure the effect of walking speed on preferred stiffness. At the start of the first experimental session, subjects completed three standard overground 10 Meter Walk Test (10MWT) trials [56] with their daily-use prosthesis. After replacing the daily-use prosthesis with the VSPA Foot and measuring preference at the three treadmill speeds, we conducted an additional three 10MWT trials at each of five different stiffness values, for a total of 15 trials. Prior to each of these trials, VSPA stiffness was remotely set to one of the five stiffness values surrounding each subject’s preferred stiffness at the self-selected treadmill speed (+ 0%, ± 15%, ± 30% deviation from preferred stiffness). Average walking speed, which we calculated as total travel distance divided by travel time as measured with a manual stopwatch, was normalized for each subject to their average 10MWT velocity with their daily-use prosthesis.

### Statistical analysis

All statistical analyses were performed in Matlab. We hypothesized that preferred stiffness varies with treadmill walking speed and body-mass. To evaluate this hypothesis, we first assessed a linear fit between walking speed and preferred stiffness, using a first-order linear mixed effects model (LMEM). When this model did not show significance, we assessed a quadratic fit, using a second-order LMEM. A simple linear regression was used to evaluate the relationship between body mass and preferred stiffness.

To evaluate our second hypothesis—that biomechanical, metabolic, and performance-based descriptors vary significantly across prosthetic ankle stiffness levels, and are locally maximized or minimized at or near the preferred stiffness—each outcome measure was treated as a dependent variable and analyzed separately using a second-order LMEM. For a descriptor to be considered a potential indicator of preference, it was necessary that it (i) show a significant second order trend with preference-normalized VSPA stiffness, and (ii) have a predicted vertex at or near the preferred stiffness (within 10% of preferred stiffness). We chose a threshold of 10% as this approximates the difference in mechanics that stem from a change in prosthesis “category” for common commercially available prosthetic feet [57]. On the basis of existing literature and preliminary studies, we identified 25 primary biomechanical descriptors of interest for potential associations with preferred stiffness (Table 1). These primary descriptors were selected for hypothesis testing from a more comprehensive set of biomechanical features (see Additional file 1: Table S1 for secondary descriptors). Preference-normalized stiffness and treadmill walking speed (relative to self-selected) were included in the model as fixed effects, with an additional random intercept per subject, and subject-specific random slopes for treadmill walking speed. For each descriptor, we assessed significance of the first and second-order fixed effect coefficients, to determine (i) if any significant second order effects were present, and (ii) if the models had an identifiable local extremum at or near the preferred stiffness. The level of significance was set at α = 0.05, with Bonferroni corrections made to reduce the total false positive rate for the 25 tested statistical tests performed. For descriptors that showed a quadratic relationship (*p* < 0.05 for the second order coefficient), we used the delta method to generate a 95% confidence interval for VSPA stiffness at which model’s vertex was located. After the second-order LMEM failed to show a significant quadratic relationship between overground self-selected walking speed and VSPA stiffness, we carried out additional post-hoc t-tests to further understand the effect of stiffness on self-selected walking speed.

## Results

### Preferred stiffness was not linearly related to treadmill speed

The first-order linear mixed effects model (LMEM) showed no significant linear relationship between walking speed and preferred stiffness (*p* = 0.081). However, a post-hoc quadratic LMEM did show a significant second-order effect of treadmill speed (*p* = 0.0046), indicating an underlying nonlinear relationship between walking speed and preference (Fig. 2B). Inter-subject mean preferred stiffness was lowest at the self-selected walking speed, compared to the fast walking speed (+ 30% self-selected) and the slow walking speed (− 30% self-selected). Intra-subject mean preferred stiffness was lowest at the self-selected walking speed for six of the seven experimental subjects (Fig. 2C).

### Heavier subjects did not prefer higher stiffness values

**Fig. 3:**
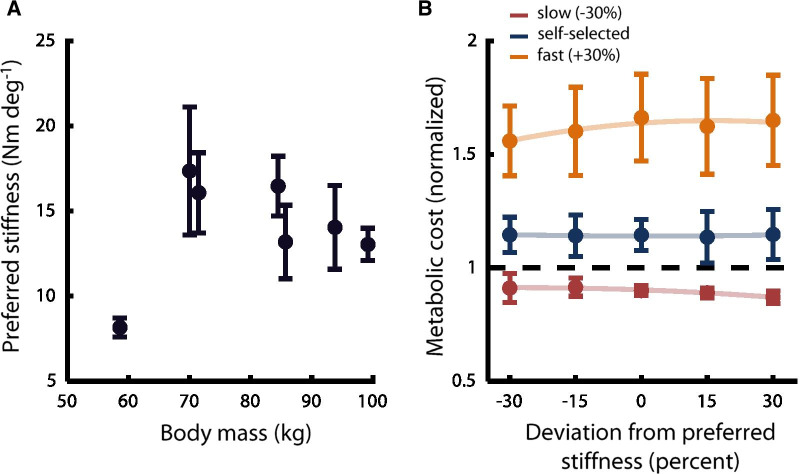
Potential anthropomorphic and metabolic factors. **A** Preferred VSPA stiffness versus body mass. Across all subjects, no significant linear trend was identified (*p* = 0.615). Points show intra-subject mean preferred stiffness across all trials and speeds. Error bars show SEM. **B** Metabolic cost versus VSPA stiffness. Metabolic expenditure did not change significantly with VSPA stiffness (LMEM, *p* > 0.5). As expected, metabolic cost was significantly different (*p* < 0.0001) at the self-selected, slow, and fast walking speeds. Each subject’s metabolic cost values are normalized to the cost measured while they walked on their daily-use prosthesis at their self-selected speed. Stiffness values are normalized to each subject’s preferred stiffness at each speed. Light lines show second-order fits to the inter-subject mean metabolic cost for each stiffness and speed (plotted points). Error bars indicate SEM

Despite the importance of body habitus as a prescriptive indicator for prosthetic stiffness [38], we did not observe a significant linear relationship between body mass and preferred stiffness (R^2^ = 0.05, *p* = 0.615, Fig. 3A).

### Preferred stiffness did not optimize metabolic rate

There was no observable linear (LMEM, *p* = 0.512) or quadratic (LMEM, *p* = 0.56) effect of preference-normalized VSPA stiffness on metabolic rate (Fig. 3B). We also did not observe significant linear (LMEM, *p* = 0.76) or quadratic (LMEM, *p* = 0.78) trends in metabolic rate as a function of weight-normalized VSPA stiffness. These results indicate that metabolic rate is not measurably affected by stiffness changes on the scale considered in this study, independent of subject preference. Treadmill speed had a significant linear effect on metabolic cost (LMEM, *p* < 0.0001).

### Preferred stiffness maximized ankle kinematic symmetry

**Fig. 4:**
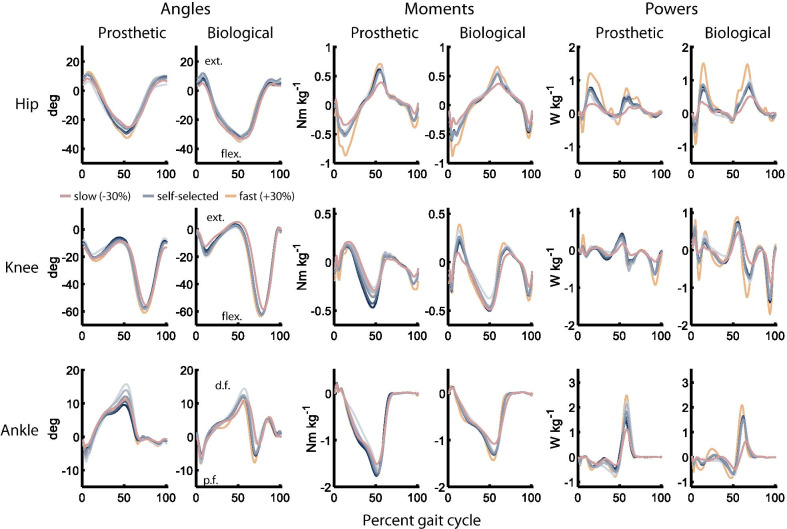
Gait biomechanics at different stiffnesses and speeds. Lines represent inter-subject average trajectories across all experimental subjects. Line shade indicates VSPA stiffness (darker is stiffer). Only the preferred stiffness trajectory is shown for the slow and fast speeds. For all joints, the positive direction is extension (ext.)/dorsiflexion (d.f.) and the negative direction is flexion (flex.)/plantar flexion (p.f.) All trajectories are time-normalized to percent gait cycle

**Fig. 5:**
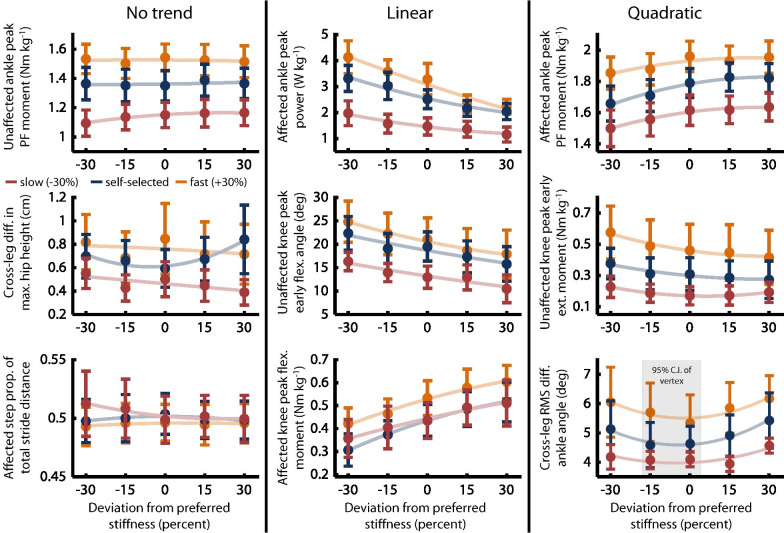
Types of trends observed in biomechanical descriptors. Our analysis showed four types of relationships between preference-normalized VSPA stiffness and different biomechanical descriptors: no trend, linear trend, quadratic trend with vertex far from the preferred stiffness, and quadratic trend with vertex at or near the preferred stiffness. Light lines show second-order fits to the inter-subject mean value of the plotted descriptor at each stiffness and speed (plotted points). All stiffness values are normalized to each subject’s preferred stiffness at each speed. Grey shading indicates the 95% CI for the stiffness value corresponding to the descriptor’s vertex, shown only when that CI includes the preferred stiffness. Error bars indicate SEM

The objective of this analysis was to identify biomechanical descriptors with an extremum at or near the preferred stiffness, which would indicate a potentially substantive role in the underlying determination of user preference. Joint angle trajectories and net joint moments were noticeably impacted by both speed and stiffness (Fig. 4), with Table 1 showing significance of the LMEM’s first- and second-order terms. Ten of the primary biomechanical predictors showed no trend (*p* > 0.05 for both terms), with nine showing a linear trend (*p* < 0.05 for the first-order term), and six showing a quadratic trend (*p* < 0.05 for the second-order term, or for both the first- and second-order terms). Second-order polynomial fits to the inter-subject mean values for a subset of these descriptors highlight each type of trend (Fig. 5).

**Fig. 6:**
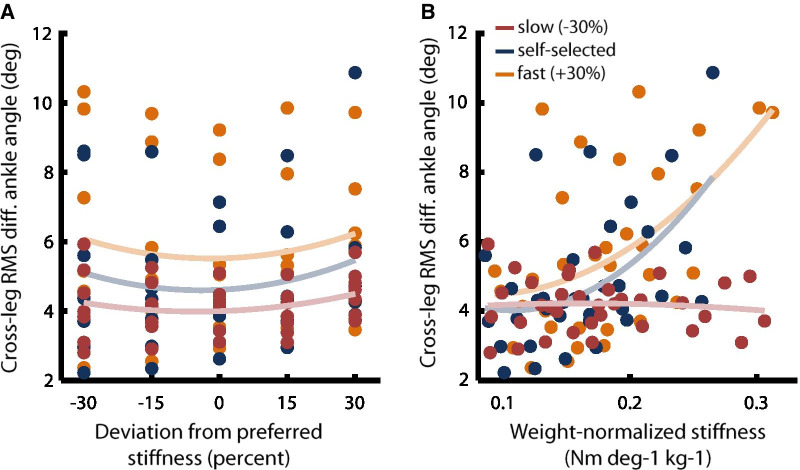
Underlying trends elucidated by preference. Normalization of stiffness values by preference highlights underlying relationships between kinematic symmetry and VSPA stiffness, which are obscured when stiffness is normalized by weight. **A** *Cross-leg RMS diff. ankle angle* versus preference-normalized stiffness. Intra-subject averages for each speed and stiffness are plotted as individual points. Light lines show a second-order fit to all pooled intra-subject averages at each speed. **B** *Cross-leg RMS diff. ankle angle* versus weight-normalized stiffness. Intra-subject averages for each speed and stiffness are plotted as individual points. Light lines show a second-order fit to all pooled intra-subject averages at each speed

Only a single descriptor—*Cross-leg RMS diff. ankle angle*, which describes stance-phase kinematic symmetry between affected and unaffected ankle joints—had a predicted vertex within 10% of the preferred stiffness (LMEM, *p* = 0.0009; vertex at 7.02% deviation from preferred stiffness). Notably, there was no significant second-order trend between Cross-leg RMS diff. ankle angle and *weight*-normalized stiffness (LMEM, *p* = 0.45), indicating that preference illuminates this underlying relationship between ankle kinematic symmetry and prosthetic foot stiffness (Fig. 6).

### Self-selected walking speed was reduced at stiffness values below the preferred

**Fig. 7:**
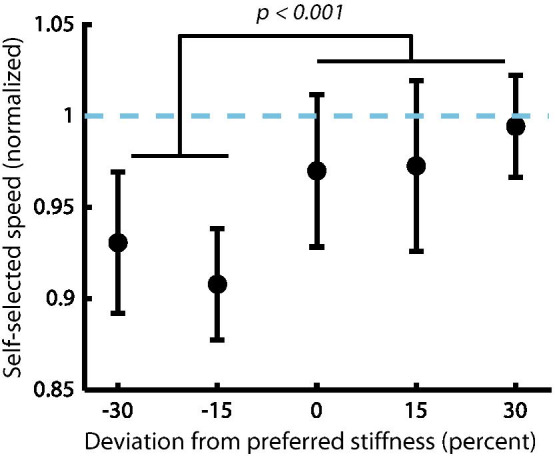
Self-selected overground walking speed at different VSPA stiffness values. Subjects walked significantly faster when VSPA stiffness was set to values at or above their preferred stiffness. Walking speeds are normalized to each subject’s self-selected overground speed while walking on their daily-use prosthesis. Inter-subject average speeds are plotted. Error bars show SEM

We did not observe a significant second-order relationship between self-selected overground walking speed and preference-normalized VSPA stiffness (LMEM, *p* = 0.51). However, we did observe a significant first-order relationship (*p* < 0.0001), and a post-hoc analysis showed that subjects chose to walk at slower speeds when VSPA stiffness was below their preferred stiffness values (t-test, *p* < 0.0001, Fig. 7).

## Discussion

In this study, we evaluated anthropomorphic, metabolic, biomechanical, and performance-based correlates of user-preferred prosthetic ankle stiffness. Our objective was to elucidate factors that users may perceive when selecting their preferred stiffness, which is an essential step toward formal incorporation of user preference in evaluating clinical assistive technologies. We found that preferred stiffness does not change linearly with walking speed; instead, subjects consistently preferred lower stiffness values at the self-selected speed. We also did not find a significant relationship between body mass and preferred stiffness. Metabolic expenditure was not measurably affected by prosthetic ankle stiffness within the experimental range. Several biomechanical descriptors showed significant trends with prosthetic ankle stiffness; however only a single measure of kinematic symmetry had an extremum near the preferred stiffness. Performance in the 10MWT improved significantly at stiffness values at or above the preferred stiffness.

We were intrigued by the nonlinear relationship between treadmill walking speed and preferred stiffness (Fig. 2), in light of the documented positive linear relationship between ankle quasi-stiffness and gait speed in persons with two unaffected biological limbs [58]. This trend was consistent across individuals; the mean preferred stiffness was lowest at the self-identified “comfortable,” intermediate speed for all but one of the experimental subjects. One possible explanation for this result is that ankle behavior has been reported to be most spring-like at intermediate walking speeds; during slow and fast walking, the unaffected biological ankle dissipates and generates energy respectively [59]. Due to its quasi-passive design, the VSPA Foot is not capable of performing net positive work, or of dissipating energy beyond the losses intrinsic to its design. As such, it is possible that subjects preferred stiffer settings at the fast and slow speeds because these settings may reduce the transfer of energy to and from the ankle joint. In other words, although lower stiffness values provide increased energy storage capacity and improved shock attenuation over stiffer joints, these potential benefits may be outweighed by feelings of instability or lack of confidence at walking speeds that are uncomfortably fast or slow. Further exploration of these relationships will be the focus of future experiments.

The stark lack of correlation we observed between preferred stiffness and body mass reinforces results from our previous studies, showing that body mass is a poor predictor of preference [39, 40]. Because user weight plays such a prominent role in the initial prosthetic prescription process, these results have particularly pressing implications for prosthetists and manufacturers. That there is not a positive, linear relationship between body mass and preference implies that other clinical tools may prove more useful than weight when designing and prescribing prostheses that people like to use.

We did not observe a significant effect of prosthetic ankle stiffness on metabolic expenditure, even when we normalized stiffness by each subject’s preference. While it is known that metabolic cost is not highly sensitive to prosthesis mechanics during level walking [41, 60–66], these previous studies have assessed metabolic cost either as a function of *weight-*normalized prosthetic joint stiffness, or of categorical stiffness [64]. Given that we have consistently shown that there is no clear linear relationship between weight and preferred stiffness, it is possible that weight-normalization may have obscured any underlying effects, by not adequately aligning the minima of individual subjects’ energy landscapes [40]. As such, we posited that preference normalization might reveal an energetic minimum at each subject’s preferred stiffness, as the vertex of an underlying quadratic relationship between preference-normalized stiffness and metabolic cost. This hypothesis was drawn from a large body of work showing that, in manipulated environments with altered dynamics, humans with two intact biological limbs subconsciously adapt their gait in ways that reduces the metabolic cost of ambulation [67–69]. Recent work has also suggested that persons with mobility impairment will sometimes adapt their gait in ways that improve the energy economy of locomotion [70].

Despite these prior results supporting our hypothesis, our analysis showed that metabolic cost was essentially unaltered in the tested range of stiffness levels. This implies that metabolic expenditure is unlikely to be a driving influence in user selection of preferred prosthetic ankle stiffness. However, these results cannot be generalized to describe how metabolism influences preference in assistive devices that have significant metabolic impact.

We also sought simple biomechanical descriptors that correlate with preference, which may provide insight into the drivers of patient preference. Identifying these descriptors could be valuable for designing new prostheses, and for developing clinical tools to quickly optimize prosthesis behavior based on inferred preference, especially for patients who are unable to quickly develop or communicate preferences. This part of the study was exploratory by nature, and further studies with more subjects will be required to confirm the importance of these parameters to the user. Our assessment revealed four distinct types of relationships between the evaluated biomechanical descriptors and prosthetic ankle stiffness. For many descriptors, we observed no significant trend, indicating that these descriptors were not consistently or substantially affected by preference-normalized stiffness, and were therefore unlikely to play a dominant role in dictating preferred VSPA stiffness. Many other descriptors trended linearly with preference-normalized stiffness; although these descriptors were affected by stiffness, they were either not maximized/minimized at the preferred stiffness, or an “optimal,” absolute (non-relative) value of the descriptor exists but was not obvious or known (e.g., is there a “most desirable” absolute peak knee flexion moment?) The third category includes those descriptors that showed a quadratic trend, with a vertex predicted at stiffness settings higher or lower than the preferred stiffness. Because descriptors in this category had extrema far from the preferred stiffness, they are unlikely to hold substantial influence in the selection of preference. The final category, which was the target of our analysis, includes descriptors that have a significant second-order relationship with preference-normalized stiffness, *and* a predicted vertex near the preferred stiffness; such predictors may be more heavily weighted by users in determining preference and available to clinicians seeking simple biomechanical goals. Only *Cross-leg RMS diff. ankle angle* fell into this category.

*Cross-leg RMS diff. ankle angle* describes the kinematic asymmetry between a subject’s prosthetic and unaffected ankle joints. Although the underlying second-order relationship between kinematic asymmetry and prosthetic joint stiffness was not unexpected, due to the sensitive linear relationship between prosthetic-side kinematics and stiffness [60, 66], the vertex’s correlation with preference provides preliminary evidence that symmetric ankle kinematics may be important to patients. A possible explanation is that ankle kinematics are closely related to the progression of the center of mass; prosthesis users may desire symmetric energy transfer between ankle elastic energy and center of mass potential energy, which they can sense via vestibular, proprioceptive, or cutaneous cues. Gait asymmetry is known to have substantial long-term ramifications for persons with amputation, with strong ties to knee osteoarthritis and other downstream effects [29, 30, 71, 72]. It is also worth noting that abnormal ankle kinematics can be readily assessed in the clinic, and are a known indicator of improper prosthetic stiffness [38]. However, the kinematic changes associated with the range of stiffness values assessed in this study are subtle, and difficult to observe with the untrained eye (Additional file 3: Movie S2). The hypothesis that preference encodes positive health-related outcomes is also suggested by the improvements we observed in 10MWT performance at stiffness values at or above the preferred stiffness. In addition, our results did not show associations between the preferred stiffness and known *negative* outcomes, such as elevated metabolic cost, reduced 10MWT performance, or increased pelvic tilt. In future longitudinal studies, we will directly measure the long-term health implications of prostheses that are designed and controlled according to user preference.

It is noteworthy that the optimum in *Cross-leg RMS diff. ankle angle* was only observed when viewed through the lens of preference-normalized stiffness (Fig. 6). This result highlights the potential for rigorous quantification of patient preference to illuminate underlying inter-subject biomechanical trends that are not otherwise visible. Additionally, although this trend was *qualitatively* consistent within the majority of individual subjects, aggregate data from all subjects were necessary to identify the significant second-order relationship between preference-normalized stiffness and *Cross-leg RMS diff. ankle angle*. The value of this group-level information lies in its potential to improve the efficiency of the prescription process, during which resource limitations (e.g. prosthetist time, prosthetic hardware, etc.) may preclude the level of patient-specific preference optimization performed in our study. Additionally, because we have shown that aggregate patient preference may correlate with improvements in walking performance according to relatively “accepted” metrics such as self-selected walking speed, providers may be more inclined to include patient feedback in the prescription process.

This study was limited by several practical considerations inherent to the VSPA Foot. We chose to use the VSPA Foot for our experiments because it is capable of producing a wide-range of highly-repeatable mechanics along a single, intuitive, continuous axis, in a lighter package than powered prostheses. However, the geometry of the foot and the overall shape of the torque–angle relationship did not perfectly reflect the behavior of typical passive prostheses. In addition, although none of the subjects preferred stiffness values at or above the VSPA’s maximum stiffness, two subjects did prefer stiffness values (at the fast speed) that were within 30% of the maximum, such that we were forced to restrict the range of experimental stiffness values to less than + 30%. Our results also showed that metabolic cost was generally higher for the VSPA Foot than for the daily-use prosthesis, and 10MWT speed generally slower. Although this is likely attributable to experience and training, it may also point to limitations of the experimental device.

Our analysis in this study was focused to a subset of all the possible factors that may contribute to user preference. For instance, we did not record electromyography, which would have provided targeted estimates of muscle activity, or upper body kinematics, which would have enabled center of mass calculations. We also did not take comprehensive surveys of patient-reported comfort and stability. Further, with robotic or quasi-passive devices that vary other parameters (e.g., variable-damping prostheses), prosthesis users may prioritize a different combination of factors, and outcome measures may be more or less sensitive. As such, our results are not intended to be comprehensive; rather they provide preliminary evidence that preference is associated with some measurable physiological factors (and not others), and highlight the importance of further research in this area.

The subjects in this study were not representative of the population of persons with below-knee amputation, due primarily to the amount of walking required for participation. However, these subjects were representative of community ambulators, which are the target population for most ankle–foot prostheses. Although it is possible that preference manifests differently in people who ambulate at lower activity levels, this falls outside the scope of the present study. Additionally, the relative number of men and women (6M, 1F) in our sample is not representative; unfortunately, we were not able to find a more balanced cohort within a reasonable time frame. Finally, adaptation times may not have been long enough to allow preferences to settle, and both preferences and gait biomechanics may continue to refine over much longer time periods.

Rigorously-measured user preference has the potential to inform the design, validation, and prescription of clinical assistive devices that increase user satisfaction and improve health outcomes. Our study sheds light on the factors that contribute to preference, which is an essential first step toward understanding its potential role in clinical practice. User preference is not a perfect metric, in that it is noisy and unlikely to encode all possible factors that determine the efficacy of a clinical device; however, it does provide a direct measurement of what users want. Additionally, we have shown its correlation with potentially meaningful biomechanical and performance-based outcomes. In the future, our methodology for measuring preference may provide a rapid means of selecting design or control parameters from a multi-dimensional space, which currently poses a substantial challenge for the field. For this to be possible, new techniques for efficiently optimizing for user preference will need to be developed [42, 43], and combined with new robotic tools that enable high-fidelity emulation of adjustable mechanical parameters [73].

## Conclusion

Our objective in this study was to understand the biomechanical indicators and potential clinical benefits of patient-preferred prosthetic ankle stiffness. We found that preferred stiffness was lowest at the self-selected treadmill speed, and that metabolic cost of walking was not minimized at the preferred stiffness, but ankle kinematic symmetry was maximized. Our results showed that self-selected overground walking speed was highest at stiffnesses at or above the preferred stiffness. This study introduces a formalized approach to identifying the metabolic and biomechanical descriptors that contribute to patient preference in design and control of assistive technology. When applied more broadly, this approach opens the door to a new preference-driven paradigm for development and prescription of rehabilitation devices.

## Supplementary Information

**Additional file 1: Table S1.** Secondary biomechanical descriptors and their trends with stiffness

**Additional file 2: Movie S1.** Design and function of the VSPA Foot

**Additional file 3: Movie S2.** Sample gait kinematics across VSPA stiffness values

## Data Availability

All data necessary to evaluate the conclusions of this manuscript are available in the paper or the additional files.
