# Supplementary material for: Understanding patient preference in prosthetic ankle stiffness
Source: J Neuroeng Rehabil. 2021 Aug 25;18:128. doi: 10.1186/s12984-021-00916-1 (PMC8390224; doi:10.1186/s12984-021-00916-1)
Supplement: Supplementary file 1 — Additional file 1: Table S1. Secondary biomechanical descriptors and their trends with stiffness [file 12984_2021_916_MOESM1_ESM.pdf]

Table S1. Secondary biomechanical descriptors and their trends with stiffness. Following completion of the primary analyses detailed in Table 1, seventy-two additional secondary descriptors were evaluated for first- and second-order fit with preference-normalized VSPA stiffness. This additional analysis was performed for completeness, to avoid missing any potential underlying trends; as such, these secondary descriptors are extensions of the primary descriptors to all joints of the affected and unaffected limbs. The table shows *p*-values for the linear (p_L) and quadratic (p_Q) terms of the LMEM fit to each descriptor. For descriptors with $p_Q < 0.05$, the lower (v_{lb}) and upper (v_{ub}) bounds on the 95% CI for the vertex stiffness (percent deviation from preferred) are also shown. * indicates significance at the α = 0.05 level (Bonferroni correction for 97 total primary and secondary hypotheses).

		p_L	p_Q	v_{lb}	v_{ub}
Hip	**Cross-leg diff. in hip ROM**	0.227	0.361	—	—
	**Cross-leg diff. in hip ROM**	0.227	0.361	—	—
	**Cross-leg diff. in peak hip ext. moment**	0.534	0.990	—	—
	**Cross-leg diff. in peak hip flex. moment**	0.001	0.918	—	—
	**Cross-leg diff. in peak hip power**	0.530	0.454	—	—
	**Cross-leg diff. in min. hip power**	0.440	0.607	—	—
	**Cross-leg RMS diff. hip power**	0.299	0.597	—	—
	**Affected hip peak ext. angle**	0.515	0.412	—	—
	**Unaffected hip peak ext. angle**	7e-6*	0.719	—	—
	**Affected hip peak ext. moment**	0.938	0.904	—	—
	**Unaffected hip peak ext. moment**	0.041	0.910	—	—
	**Affected hip peak power**	0.202	0.354	—	—
	**Unaffected hip peak power**	0.018	0.925	—	—
	**Unaffected hip peak early ext. moment**	0.274	0.258	—	—
	**Affected hip peak early ext. moment**	0.913	0.951	—	—
	**Affected hip peak flex. angle**	4e-4*	0.553	—	—
	**Unaffected hip peak flex. angle**	0.758	0.855	—	—
	**Affected hip peak flex. moment**	0.105	0.977	—	—
	**Unaffected hip peak flex. moment**	0.012	0.246	—	—
	**Affected hip min. power**	0.481	0.995	—	—
	**Unaffected hip min. power**	0.258	0.410	—	—
	**Affected hip peak early flex. angle**	4e-10*	0.853	—	—
	**Unaffected hip peak early flex. angle**	0.307	0.982	—	—
	**Cross-leg diff. in peak early hip flex. angle**	0.573	0.588	—	—
	**Affected hip ROM**	3e-4*	0.985	—	—
	**Unaffected hip ROM**	4e-4*	0.700	—	—
	**Affected hip moment range**	0.456	0.955	—	—
	**Unaffected hip moment range**	0.001	0.408	—	—
	**Affected hip power range**	0.514	0.507	—	—
	**Unaffected hip power range**	0.062	0.551	—	—
Knee	**Cross-leg diff. in knee ROM**	0.030	0.422	—	—
	**Cross-leg diff. in peak knee flex. moment**	0.177	0.060	—	—
	**Cross-leg RMS diff. knee power**	0.001*	0.092	—	—
	**Affected knee peak ext. angle**	8e-12*	0.819	—	—

	**Unaffected knee peak ext. angle**	0.104	0.685	—	—
	**Unaffected knee peak ext. moment**	1e-4*	0.010	2.8	51.7
	**Affected knee peak power**	5e-6*	0.535	—	—
	**Unaffected knee peak power**	3e-7*	0.083	—	—
	**Affected knee peak early ext. moment**	1e-4*	0.485	—	—
	**Affected knee peak flex. angle**	2e-17*	0.258	—	—
	**Unaffected knee peak flex. angle**	0.136	0.856	—	—
	**Unaffected knee peak flex. moment**	0.352	0.584	—	—
	**Affected knee min. power**	0.066	0.797	—	—
	**Unaffected knee min. power**	0.001	0.169	—	—
	**Affected knee peak early flex. angle**	0.001	0.648	—	—
	**Unaffected knee peak early flex. angle**	6e-4*	0.426	—	—
	**Cross-leg diff. in peak early knee flex. angle**	0.002	0.495	—	—
	**Affected knee moment range**	0.001	0.842	—	—
	**Unaffected knee moment range**	0.003	0.005	1.0	37.0
	**Affected knee power range**	0.451	0.993	—	—
	**Unaffected knee power range**	4e-6	0.075	—	—
	**Cross-leg diff. in ankle ROM**	4e-20*	0.492	—	—
	**Cross-leg diff. in peak ankle DF moment**	0.309	0.823	—	—
	**Cross-leg diff. in min. ankle power**	0.210	0.175	—	—
	**Cross-leg RMS diff. ankle power**	1e-6*	0.094	—	—
	**Unaffected ankle peak DF angle**	0.064	0.910	—	—
	**Affected ankle peak DF moment**	0.571	0.818	—	—
	**Unaffected ankle peak DF moment**	2e-7*	0.069	—	—
	**Unaffected ankle peak power**	0.875	0.492	—	—
	**Unaffected ankle peak early DF moment**	6e-8*	0.091	—	—
	**Affected ankle peak early DF moment**	0.609	0.841	—	—
**Ankle**	**Affected ankle peak PF angle**	3e-24*	0.025	13.6	200
	**Unaffected ankle peak PF angle**	0.417	0.910	—	—
	**Affected ankle min. power**	2e-16*	0.340	—	—
	**Unaffected ankle min. power**	0.588	0.945	—	—
	**Affected ankle peak early PF angle**	2e-23*	0.031	9.7	207.1
	**Unaffected ankle peak early PF angle**	0.312	0.599	—	—
	**Cross-leg diff. in peak early ankle PF angle**	0.852	0.223	—	—
	**Affected ankle moment range**	5e-5*	0.161	—	—
	**Unaffected ankle moment range**	0.188	0.617	—	—
	**Affected ankle power range**	3e-19*	0.284	—	—
	**Unaffected ankle power range**	0.830	0.533	—	—
